# Genetic parameters for various semen production and quality traits and indicators of male and female reproductive performance in Nellore cattle

**DOI:** 10.1186/s12864-023-09216-5

**Published:** 2023-03-27

**Authors:** Felipe E. Carvalho, José Bento S. Ferraz, Victor B. Pedrosa, Elisangela C. Matos, Joanir P. Eler, Marcio R. Silva, José D. Guimarães, Fernando O. Bussiman, Barbara C. A. Silva, Fernando A. Cançado, Henrique A. Mulim, Rafael Espigolan, Luiz F. Brito

**Affiliations:** 1 Department of Veterinary Medicine, Faculty of Animal Science and Food Engineering, University of São Paulo, Pirassununga, SP Brazil; 2 Department of Animal Sciences, Purdue University, 270 S. Russell Street, West Lafayette, IN 47907 USA; 3 Department of Veterinary Medicine, Federal University of Vicosa, Vicosa, MG Brazil; 4 Department of Animal and Dairy Science, University of Georgia, Athens, GA USA

**Keywords:** Fertility, Genetic correlation, Heritability, Precocity, Reproduction, Sex-dependent, Zebu

## Abstract

**Background:**

Given the economic relevance of fertility and reproductive traits for the beef cattle industry, investigating their genetic background and developing effective breeding strategies are paramount. Considering their late and sex-dependent phenotypic expression, genomic information can contribute to speed up the rates of genetic progress per year. In this context, the main objectives of this study were to estimate variance components and genetic parameters, including heritability and genetic correlations, for fertility, female precocity, and semen production and quality (andrological attributes) traits in Nellore cattle incorporating genomic information.

**Results:**

The heritability estimates of semen quality traits were low-to-moderate, while moderate-to-high estimates were observed for semen morphological traits. The heritability of semen defects ranged from low (0.04 for minor semen defects) to moderate (0.30 for total semen defects). For seminal aspect (SMN_ASPC) and bull reproductive fitness (BULL_FIT), low (0.19) and high (0.69) heritabilities were observed, respectively. The heritability estimates for female reproductive traits ranged from 0.16 to 0.39 for rebreeding of precocious females (REBA) and probability of pregnancy at 14 months (PP14), respectively. Semen quality traits were highly genetically correlated among themselves. Moderate-to-high genetic correlations were observed between the ability to remain productive in the herd until four years of age (stayability; STAY) and the other reproductive traits, indicating that selection for female reproductive performance will indirectly contribute to increasing fertility rates. High genetic correlations between BULL_FIT and female reproductive traits related to precocity (REBA and PP14) and STAY were observed. The genetic correlations between semen quality and spermatic morphology with female reproductive traits ranged from -0.22 (REBA and scrotal circumference) to 0.48 (REBA and sperm vigor). In addition, the genetic correlations between REBA with semen quality traits ranged from -0.23 to 0.48, and with the spermatic morphology traits it ranged from -0.22 to 0.19.

**Conclusions:**

All male and female fertility and reproduction traits evaluated are heritable and can be improved through direct genetic or genomic selection. Selection for better sperm quality will positively influence the fertility and precocity of Nellore females. The findings of this study will serve as background information for designing breeding programs for genetically improving semen production and quality and reproductive performance in Nellore cattle.

**Supplementary Information:**

The online version contains supplementary material available at 10.1186/s12864-023-09216-5.

## Background

Fertility and reproductive performance directly impact the beef cattle industry's profitability [1]. Numerous environmental effects commonly influence fertility and reproductive traits (e.g., diet and nutritional management, climatic conditions, year season, reproductive management) [2–4], which are genetically controlled by many genes with small effects [5–8]. Reproduction outcomes depend on the fertility levels of both males and females and their potential interactions [9]. For instance, semen quality and quantity has been shown to directly affect fertility and conception rates [10–12].

Improving the quality and quantity of semen produced by genetically superior bulls will increase the availability of semen from elite bulls at a reasonable price to farmers [13] and speed up the rates of genetic progress in the national beef cattle populations. Therefore, genetic selection for semen quality will contribute to better conception rates and lower reproductive costs per progeny [12], improving the beef cattle industry’s sustainability. Considering the economic relevance of reproductive efficiency, various studies have investigated the genetic background of fertility and reproductive traits in cattle [8, 14–17]. However, most studies were done in taurine (*Bos taurus taurus*) breeds without incorporating genomic information. Furthermore, there is a lack of estimates of genetic correlations between male and female fertility and reproduction traits, especially in Zebu (*Bos taurus indicus*) breeds such as Nellore, one of the most important beef cattle breeds in Brazil (one of the largest beef cattle producers in the world) [18].

The low heritability estimates of most reproductive traits, as routinely measured in worldwide breeding programs, result in reduced rates of genetic progress per generation compared to high-heritability traits [8, 19, 20]. Given the genetic complexity of fertility and reproductive traits, their sex-dependent phenotypic expression, and the availability of genomic information, it is crucial to evaluate the genetic relationship between fertility and precocity in females and semen quality and production traits of breeding bulls to improve reproductive performance in beef cattle populations. Hence, the main objectives of this study were to estimate genetic parameters, including variance components, heritabilities, and genetic correlations, for various fertility, female precocity, and semen production and quality (andrological attributes) traits in Nellore cattle (*Bos taurus indicus*) incorporating genomic information.

## Results

**Fig. 1:**
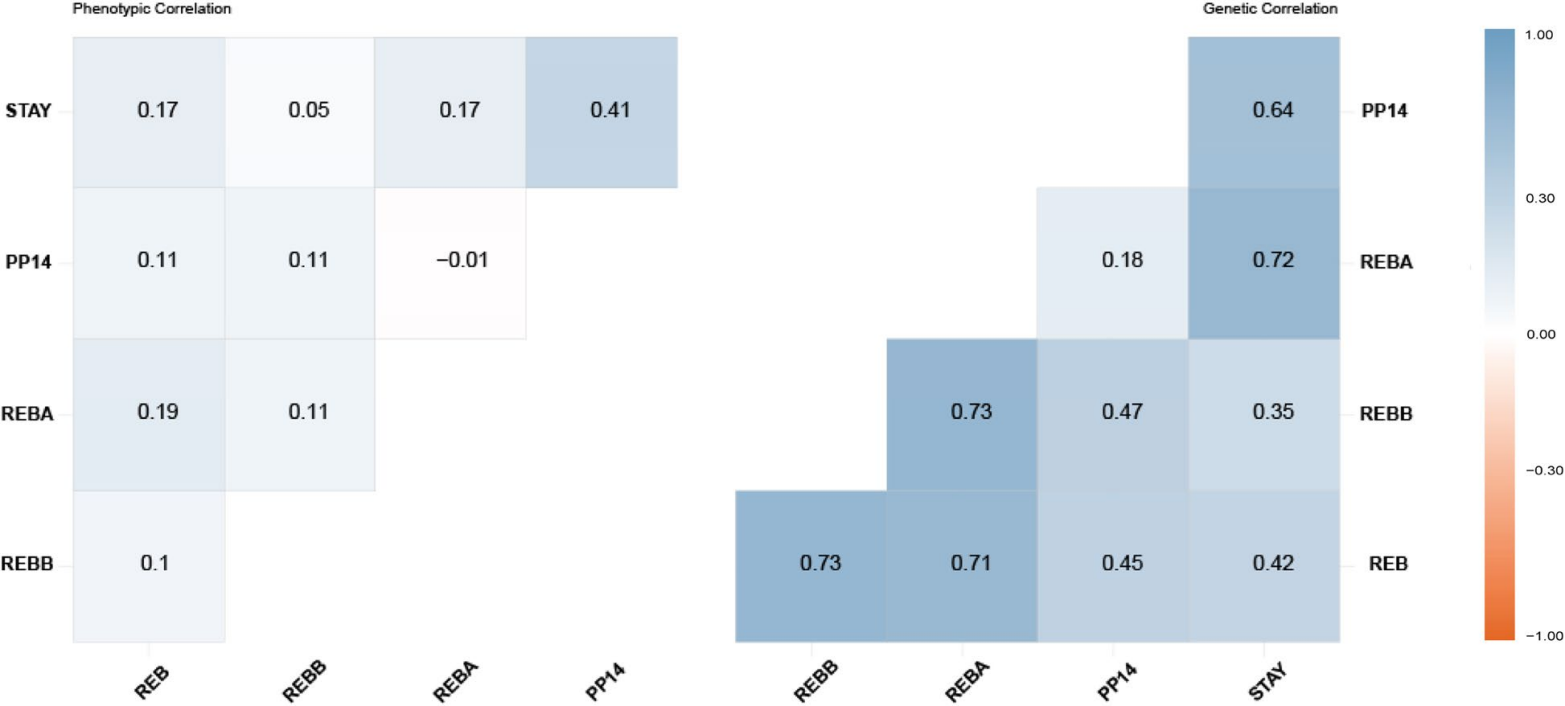
Heatmap of genetic correlation of REB: All records of rebreeding of females; REBB: Rebreeding of females that entered reproduction at two years old; REBA: Rebreeding of precocious heifers; PP14: Pregnancy probability at 14 months; STAY: Ability to remain productive in the herd. The SE for the genetic correlation estimates was < 0.001

**Fig. 2:**
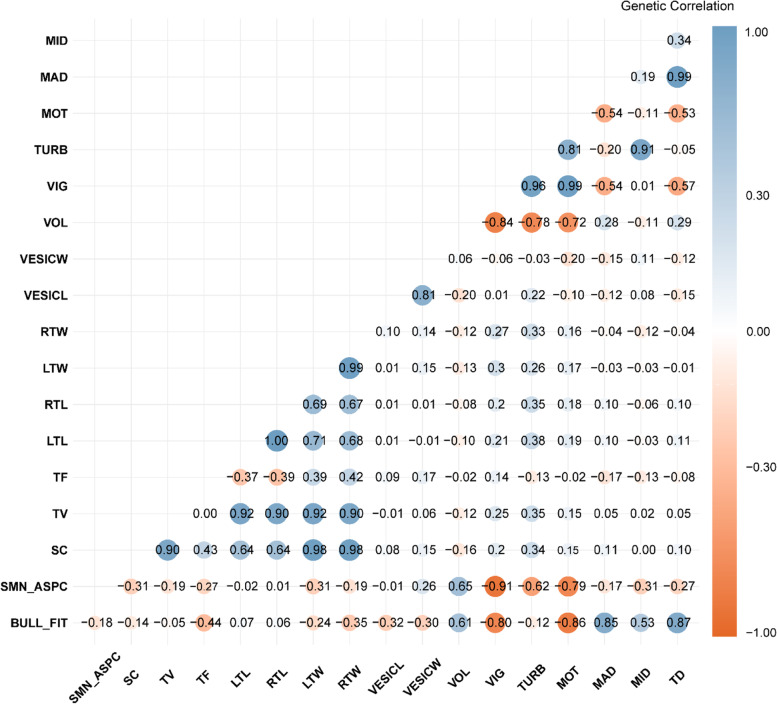
Heatmap of genetic correlations of ejaculate volume (VOL), spermatic vigor (VIG), spermatic vortex (TURB), rectilinear progressive sperm motility (MOT), scrotal circumference (SC), left testicular length (LTL), right testicular length (RTL), left testicular width (LTW), right testicular width (RTW), seminal vesicle length (VESICL), seminal vesicle width (VESICW), testicular volume (TV), testicular format (TF), percentage of sperm cells with major sperm defects (MAD), percentage of sperm cells with minor sperm defects (MID), percentage of total sperm cells with sperm defects (TD), evaluation andrological bull's fitness (BULL_FIT) and, evaluation of a seminal aspect (SMN_ASPC). The SE for the genetic correlations estimated was < 0.001

**Fig. 3:**
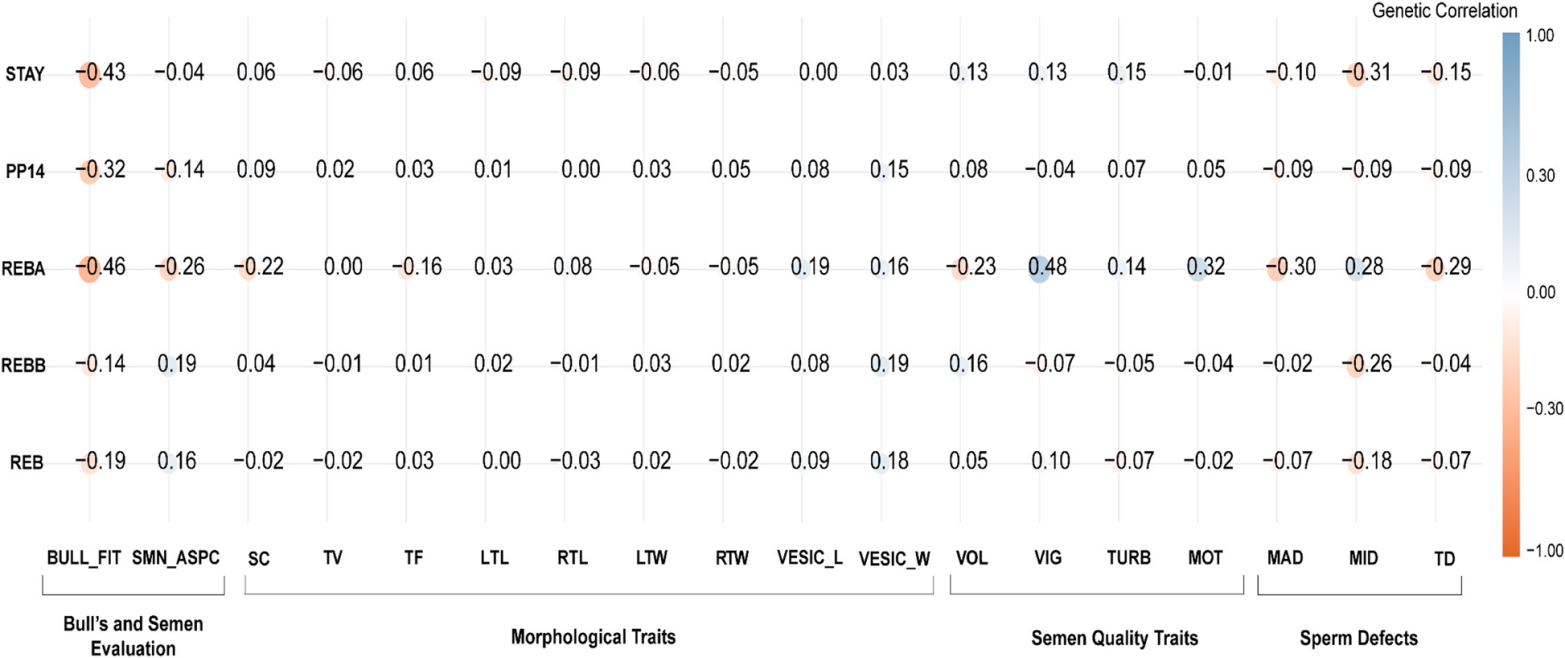
Heatmap of genetic correlation of female reproductive traits, REB: All records of rebreeding of females; REBB: Rebreeding of females that entered reproduction at two years old; REBA: Rebreeding of primiparous; PP14: Pregnancy probability at 14 months; STAY: Ability to remain productive in the herd; The male traits included ejaculate volume (VOL), spermatic vigor (VIG), spermatic vortex (TURB), rectilinear progressive sperm motility (MOT), scrotal circumference (SC), left testicular length (LTL), right testicular length (RTL), left testicular width (LTW), right testicular width (RTW), seminal vesicle length (VESICL), seminal vesicle width (VESICW), testicular volume (TV), testicular format (TF), percentage of sperm cells with major sperm defects (MAD), percentage of sperm cells with minor sperm defects (MID), percentage of total sperm cells with sperm defects (TD), evaluation andrological bull's fitness (BULL_FIT) and, evaluation of a seminal aspect (SMN_ASPC). The SE for the genetic correlation estimates was < 0.001

The descriptive statistics of the andrological traits in Nellore animals are shown in Table 1. Means of heritability estimates as well as genetic and residual variances are shown in Tables 2 and 3 for male and female reproductive traits, respectively. The estimates of genetic correlations among female reproductive traits are presented in Fig. 1, between female reproductive traits and andrological traits in Fig. 2, and between female reproductive performance and semen traits in Fig. 3. The SE for all heritability and genetic correlation estimates were lower than 0.001.

**Table 1:** Summary statistics for male and female fertility and reproduction traits in Nellore cattle

**Traits**	**N**	**Mean (SD)**	**Mode**		**Minimum**	**Maximum**	**NCG**
	**Male traits**						
	Semen quality						
**VOL (mL)**	15,882	4.07 (2.04)	-		0.50	20.00	617
**VIG (0–5)**	14,361	3.12 (0.54)	3		0	5	586
**TURB (0–5)**	14,877	1.16 (1.10)	0		0	5	608
**MOT (%)**	17,225	70.75(11.40)	-		5	95	606
	Morphological traits						
**SC (cm)**	18,435	33.00 (2,64)	-		22.50	48.00	803
**LTL (cm)**	18,693	12.08 (1.27)	-		5.20	18.50	904
**RTL (cm)**	18,680	12.11 (1.25)	-		6.70	19.00	903
**LTW (cm)**	18,677	6.53 (0.67)	-		3.00	9.30	903
**RTW (cm)**	18,662	6.58 (0.67)	-		4.00	9.50	902
**VESIC_L (cm)**	15,054	8.62 (1.93)	-		3.00	16.00	622
**VESIC_W (cm)**	15,038	2.23 (0.61)	-		0.50	10.00	622
**TV (dm**^**3**^**)**	15,659	0.25 (0.05)	-		0.06	0.49	631
**TF (1–4)**	18,848	1.85 (0.52)	2		1	4	917
	Sperm defects						
**MAD (%)**	14,312	12.75 (10.45)	-		0	131	591
**MID (%)**	13,743	4.67 (4.03)	-		0	67	517
**TD (%)**	14,621	17.33 (11.74)	-		0	136	606
	Bulls and semen evaluation						
**BULL_FIT (1–4)**	2,813	1.34 (0.74)	1		1	4	61
**SMN_ASPC (1–4)**	3,839	2.34 (0.94)	2		1	4	118
	**Female traits**						
				**%S**	1		
**REB (1–2)**	65,836	1.52 (0.50)	2	52.10	1	2	191
**REBB (1–2)**	59,675	1.55 (0.50)	2	55.00	1	2	184
**REBA (1–2)**	8,108	1.31 (0.46)	1	31.04	1	2	89
**PP14 (1–2)**	35,057	1.18 (0.39)	1	18.29	1	2	90
**STAY (1–2)**	127,106	1.27 (0.45)	1	28.59	1	2	201

*VOL* Ejaculate volume, *VIG* Spermatic vigor, *TURB* Spermatic vortex, *MOT* Rectilinear progressive sperm motility, *SC* Scrotal circumference, *LTL* Left testicular length, *RTL* Right testicular length, *LTW* Left testicular width, *RTW* Right testicular width, *VESIC_COMP*, *VESICL* Seminal vesicle length, *VESICW* Seminal vesicle width, *TV* Testicular volume, *TF* Testicular format, *MAD* Percentage of sperm cells with major sperm defects, *MID* Percentage of sperm cells with minor sperm defects, *TD* Percentage of total sperm cells with sperm defects, *BULL_FIT* evaluation andrological bull's fitness, *SMN_ASPC* Evaluation of seminal aspect, *REB* All records of rebreeding of females, *REBB* Rebreeding of females that entered reproduction at two years old, *REBA* Rebreeding of precocity heifers, *PP14* Pregnancy probability at 14 months, *STAY* Ability to remain productive in the herd, *N* Total of records, *SD* Standard deviation, *NGC* Number of contemporary groups, *%S* Percentage success rate

**Table 2:** Variance components, heritability estimates, highest posterior density range (HPD; 5%—95% interval), and Geweke score for andrological traits in Nellore cattle

**Trait**		**Mean**	**HPD Interval**		**Geweke**
			**(5% 95%)**		**(Z-score)**
	**Bull's trait**				
**VOL**	${\sigma }_{u}^{2}$	0.20	0.07	0.33	0.26
	h^2^	**0.05**	0.02	0.09	0.26
**VIG**	${\sigma }_{u}^{2}$	7.97E^−03^	3.78E^−03^	1.22E^−02^	0.21
	h^2^	**0.03**	1.63E^−02^	5.20E^−02^	0.21
**TURB**	${\sigma }_{u}^{2}$	0.03	0.01	0.06	-0.18
	h^2^	**0.04**	0.02	0.07	-0.18
**MOT**	${\sigma }_{u}^{2}$	6.71	3.39	10.03	0.03
	h^2^	**0.05**	0.03	0.08	0.03
**SC**	${\sigma }_{u}^{2}$	4.69	4.32	5.05	-0.01
	h^2^	**0.75**	0.68	0.81	0.09
**LTL**	${\sigma }_{u}^{2}$	0.26	0.21	0.30	0.03
	h^2^	**0.26**	0.22	0.30	0.03
**RTL**	${\sigma }_{u}^{2}$	0.27	0.23	0.32	-0.05
	h^2^	**0.29**	0.24	0.33	0.05
**LTW**	${\sigma }_{u}^{2}$	0.08	0.07	0.09	0.02
	h^2^	**0.30**	0.25	0.34	0.02
**RTW**	${\sigma }_{u}^{2}$	0.08	0.07	0.10	-0.04
	h^2^	**0.31**	0.26	0.35	-0.04
**VESIC_L**	${\sigma }_{u}^{2}$	0.31	0.21	0.40	0.11
	h^2^	**0.14**	0.10	0.18	0.11
**VESIC_W**	${\sigma }_{u}^{2}$	0.05	0.04	0.06	0.02
	h^2^	**0.17**	0.13	0.21	0.02
**TF**	${\sigma }_{u}^{2}$	3.47E^−04^	2.70E^−04^	4.23E^−04^	-0.01
	h^2^	**0.20**	0.15	0.24	-0.01
**TV**	${\sigma }_{u}^{2}$	3.95E^−04^	3.26E^−04^	4.63E^−04^	0.02
	h^2^	**0.32**	0.27	0.37	0.03
**MAD**	${\sigma }_{u}^{2}$	14.68	10.33	19.03	0.01
	h^2^	**0.15**	0.11	0.19	-0.01
**MID**	${\sigma }_{u}^{2}$	0.52	0.05	0.99	0.65
	h^2^	**0.04**	0.01	0.10	0.37
**TD**	${\sigma }_{u}^{2}$	35.00	19.07	50.93	0.07
	h^2^	**0.30**	0.17	0.43	0.07
**SMN_ASPC**	${\sigma }_{u}^{2}$	0.17	0.11	0.23	-0.12
	h^2^	**0.19**	0.02	0.36	-0.05
**BULL_FIT**	${\sigma }_{u}^{2}$	7.51	5.41	9.60	0.11
	h^2^	**0.69**	0.54	0.83	0.10

*VOL* Ejaculate volume, *VIG* Spermatic vigor, *TURB* Spermatic vortex, *MOT* Rectilinear progressive sperm motility, *SC* Scrotal circumference, *LTL* Left testicular length, *RTL* Right testicular length, *LTW* Left testicular width, *RTW* Right testicular width, *VESICL* Seminal vesicle length, *VESICW* Seminal vesicle width, *TV* Testicular volume, *TF* Testicular format, *MAD* Percentage of sperm cells with major sperm defects, *MID* Percentage of sperm cells with minor sperm defects, *TD* Percentage of total sperm cells with sperm defects, *BULL_FIT* evaluation andrological bull's fitness, *SMN_ASPC* Evaluation of seminal aspect, *REB* All records of rebreeding of females, *REBB* Rebreeding of females that entered reproduction at two years old, *REBA* Rebreeding of precocity heifers, *PP14* Pregnancy probability at 14 months, *STAY* Ability to remain productive in the herd. The SE for all heritability estimated was < 0.001

**Table 3:** Variance components, heritability estimates, highest posterior density range (HPD; 5%—95% interval), and Geweke score for female reproduction traits in Nellore cattle

**Trait**			**HPD Interval**		**Geweke**
		**Mean**	**(5% 95%)**		**(Z-score)**
		**Reproductive female traits**			
**REB**	${\sigma }_{u}^{2}$	0.27	0.21	0.32	-0.04
	h^2^	0.20	0.10	0.14	0.07
**REBB**	${\sigma }_{u}^{2}$	0.30	0.23	0.36	0.16
	h^2^	0.20	0.16	0.22	0.17
**REBA**	${\sigma }_{u}^{2}$	0.20	0.09	0.30	0.03
	h^2^	0.16	0.09	0.24	0.02
**PP14**	${\sigma }_{u}^{2}$	0.76	0.61	0.91	0.09
	h^2^	0.39	0.34	0.43	0.09
**STAY**	${\sigma }_{u}^{2}$	0.21	0.17	0.23	-0.04
	h^2^	0.17	0.15	0.19	-0.04

*REB* All records of rebreeding of females, *REBB* Rebreeding of females that entered reproduction at two years old, *REBA* Rebreeding of precocious heifers, *PP14* Pregnancy probability at 14 months, *STAY* Ability to remain productive in the herd. The SE for all the heritability estimates was < 0.001

### Bull breeding soundness exam traits

The semen quality traits had an average of 4.07 mL, 3.12 (score), 1.16 (score), and 70.75% for VOL, VIG, TURB, and MOT, respectively. For VIG and TURB, the measurement scale ranged from 0 to 5, and for VOL and MOT, there was a variation of 0.50 mL to 20.00 mL and 5% to 95%, respectively. For the andrological morphology trait group, an average of 30.00 cm was observed for SC, ranging from 22.50 cm to 48.00 cm, and for the testicular biometry measurements, an average length of 12.08 cm and 12.11 cm (LTL and RTL), and a width of 6.53 cm and 6.58 cm (LTW and RTW) were observed. The averages for vesicles ranged from 2.23 cm to 8.62 cm, for width and length, respectively. For TV the average were 0.25 dm^3^ and for TF the most frequent level was the long-moderate testicular class. The group of traits related to sperm defects presented averages of 12.75%, 4.67%, and 17.33% for the major and minor defects and total evaluated defects, respectively. BSE and semen evaluation with averages of 1.34 and 2.34 show a high frequency of bulls in the class suitable for reproduction and milky semen (related to higher sperm concentration).

### Female reproductive performance and precocity traits

The Nellore female rebreeding ability had a success rate of 31% for the rebreeding of heifers, 55% for the rebreeding of 2-year-old heifers, and 52.10% for general female rebreeding (REBA, REBB, and REB, respectively). The success rate for PP14 was 18.29% and for STAY was 28.59%. In general, the female rebreeding dataset showed a greater number of females in the first calving interval rest period, less than 60 days from the first parturition to the second mating or insemination, as shown in Supplementary Figure S1. For heifers (REBA), the highest proportion of animals was in classes 1 (< 60 days) and 5 (> 151) and for REBB and REB in classes 1 (< 60 days) and 2 (< 91).

### Heritability estimates for andrological traits

The heritability estimates for semen quality traits (Table 2) ranged from 0.03 to 0.05, including 0.05, 0.03, 0.04, and 0.05 for VOL, VIG, TURB, and MOT, respectively. The heritability estimates for semen biometrical traits ranged from 0.14 (VESIC_L) to 0.75 (SC). The heritability of biometrical testicular traits, i.e., LTL, RTL, LTW, and RTW, ranged from 0.29 to 0.31, followed by 0.20 and 0.32 for TF and TV, respectively. Seminal vesicle size had moderate heritability estimates of 0.14 and 0.17 for VESIC_L and VESIC_W, respectively. The heritability estimates for semen defects ranged from low for minor defects (0.04) and major defects (0.15), to moderate (0.30) for total defects (TD). For SMN_ASPC, a moderate heritability (0.19) was observed. BULL_FIT presented a high heritability estimate of 0.69.

### Heritability of female reproductive performance and precocity traits

The heritability estimates for female reproductive traits ranged from low to moderate magnitude, as shown in Table 2. Moderate heritability estimates were observed for rebreeding ability and female reproductive performance: REB (0.20), REBB (0.20), REBA (0.16), STAY (0.17), and PP14 (0.39).

### Genetic correlation between female reproductive efficiency and precocity traits

The genetic correlations between female reproductive efficiency traits are presented in Fig. 1. The female reconception traits are moderately genetically correlated, with estimates ranging from 0.71 (REB and REBA) to 0.73 (REB and REBB; REBB and REBA). However, the genetic correlations between PP14 and reconception traits were low, such as 0.18 between REBA and PP14; 0.45 between PP14 and REB; and 0.47 between PP14 and REBB. The genetic correlations between STAY and the other reproductive traits were of moderate to high magnitude, as with REB (0.42), REBB (0.35), REBA (0.72), and PP14 (0.64). These results indicate that direct selection for female reproduction traits will result in indirect improvements in other fertility traits, especially those related to reconception, female precocity, and herd stayability.

### Genetic correlation among andrological traits

The genetic correlations among andrological traits are presented in Fig. 2, including the genetic relationship among semen quality, semen morphological traits, semen defects, bulls’ breeding fitness, and overall semen evaluation. In general, semen quality traits were highly genetically correlated among themselves (Fig. 2). In contrast, high negative genetic correlations were observed between VOL and VIG (-0.84 ± 0.001), TURB ± SE (-0.78 ± 0.001), and MOT (-0.72 ± 0.001). Negative genetic correlations for VOL with testicular biometrical traits were also observed, ranging from -0.16 ± 0.002 (VOL and SC) to -0.08 ± 0.001 (VOL and RTL). However, the genetic correlations between semen quality and morphological traits were mainly positive, ranging from moderate to high magnitude (Fig. 2).

The genetic correlation between semen quality traits and sperm defects were negative and favorable between MAD with VIG, TURB, and MOT with values of -0.54 ± 0.001, -0.57 ± 0.001, -0.20 ± 0.003, respectively. The genetic correlations between MID and semen quality traits were close to zero with VIG (0.01 ± 0.003) and MOT (-0.11 ± 0.002), but high with TURB (0.91 ± 0.002; Fig. 2). The bull's breeding fitness trait and semen aspects were moderately and positively genetically correlated (VOL x BULL_FIT = 0.61 ± 0.006; VOL x SMN_ASPC = 0.65 ± 0.005). These results indicate that selection for a higher ejaculate volume can generate less suitable bulls for reproduction and a more opalescent seminal appearance. In contrast, the genetic correlation between sperm efficiency traits (VIG, TURB, and MOT) with BULL_FIT was -0.80 ± 0.004, -0.12 ± 0.012, and -0.86 ± 0.002, respectively, and with SMN_ASPC of -0.91 ± 0.003, -0.62 ± 0.005, and -0.79 ± 0.003, respectively. These results indicate that selection for bulls with higher sperm quality might result in bulls with better breeding fitness and seminal aspects. Bulls with a better breeding fitness may present better morphological conformations, as indicated by the genetic correlations between semen morphological traits and BULL_FIT, with SC (-0.17 ± 0.006), BULL_FIT with RTW and LTW (-0.24 ± 0.004 and -0.35 ± 0.005), BULL_FIT with VESIC_L and VESIC_W (-0.32 ± 0.006 and -0.30 ± 0.000), and BULL_FIT with TF (-0.44 ± 0.004). Furthermore, we observed high and positive genetic correlations between sperm defects, including MID and MAD (0.85 ± 0.005), MID and TD (0.53 ± 0.003), and MAD and TD (0.87 ± 0.001).

### Genetic correlations between female reproductive efficiency and andrological traits

The genetic correlations between female reproductive traits and semen quality, semen morphological traits, semen defects, bull's breeding fitness, and semen evaluation traits are shown in Fig. 3. Regarding the genetic correlations between male and female traits, moderate estimates were observed between BULL_FIT and traits related to precocity and female productivity, such as BULL_FIT and PP14 (-0.32 ± 0.001), BULL_FIT and REBA (-0.46 ± 0.003), and BULL_FIT and STAY (-0.43 ± 0.001).

The genetic correlations between semen quality and morphology with female reproductive traits were low (Fig. 3). The genetic relationship of REBA with semen quality traits were -0.23 ± 0.002 (VOL), 0.48 ± 0.001 (VIG), 0.14 ± 0.003 (TURB), and 0.32 ± 0.001 (MOT). The genetic correlations between STAY and semen quality traits ranged from -0.01 ± 0.000 (STAY and MOT) to 0.15 ± 0.001 (STAY and TURB). Sperm defects are favorably (but lowly) correlated with STAY, as indicated by the genetic correlations between STAY and MID (-0.31 ± 0.001), STAY and MAD (-0.10 ± 0.001), and STAY and TD (-0.15 ± 0.001). Sperm defects are also favorably correlated with female reproductive traits, such as -0.18 ± 0.001 (REB with MID), -0.26 ± 0.001 (REBB with MID), -0.30 ± 0.001 (REBA with MAD), and -0.31 ± 0.000 (STAY with MID).

## Discussion

### Male fertility and reproduction traits

The average semen quality traits (Table 4) of the bulls evaluated indicate a good reproductive potential for producing high-quality semen for artificial insemination purposes [21]. The averages observed are within the reproductive standards required by the Nellore cattle organizations in Brazil, in which MOT values are commonly expected to range from 50 to 75%, and VIG and TURB from 3 to 5 [22]. MOT is one of the most relevant semen quality traits due to its association with bull fertility [4]. Butler et al. [23] evaluated MOT before and after thawing and found values below 50% in Angus cattle. MOT has also been indicated as an essential fertility indicator in sheep and humans [24–26].

**Table 4:** Statistical models fitted for the male and female fertility and reproduction traits in Nellore cattle

Model effects	Traits
**Male traits**	
CG, IDAP, IDAP^2^, a, and e	VOL; VIG; TURB; MOT; LTL; RTL; LTW; RTW; VESIC_L; VESIC_W; TV; FT; MID; MAD; TD; SMN_ASCP; and BULL_FIT
CG, IDAP, IDAP^2^, GMAND, a, and e	SC
**Female traits**	
CG, CIR, a, and e	REB; REBA; REBB
CG, DTJN, GMAND, a, and e	PP14
CG, a, and e	STAY

*VOL* Ejaculate volume, *VIG* Spermatic vigor, *TURB* Spermatic vortex, *MOT* Rectilinear progressive sperm motility, *SC* Scrotal circumference, *LTL* Left testicular length, *RTL* Right testicular length, *LTW* Left testicular width, *RTW* Right testicular width, *VESIC_COMP*, *VESICL* Seminal vesicle length, *VESICW* Seminal vesicle width, *TV* Testicular volume, *TF* Testicular format, *MAD* Percentage of sperm cells with major sperm defects, *MID* Percentage of sperm cells with minor sperm defects, *TD* Percentage of total sperm cells with sperm defects, *BULL_FIT* evaluation andrological bull's fitness, *SMN_ASPC* Evaluation of seminal aspect, *REB* All records of rebreeding of females, *REBB* Rebreeding of females that entered reproduction at two years old, *REBA* Rebreeding of precocity heifers, *PP14* Pregnancy probability at 14 months, *STAY* Ability to remain productive in the herd, *CG* Contemporary group, *IDAP* Age measured around 15 months as a covariate, *IDAP*^*2*^ quadratic effect of age, *GMAND* is the weaning management group as an uncorrelated random effect, *CIR* Calving rest intervals, *DTJN* Calf birth date, *a* Random animal additive genetic effect, *e* residual random effect

Scrotal circumference is a predictor of fertility in bulls` daughters [27, 28]. A study based on 2,055 Nellore bulls at five different ages reported average SC ranging from 32.10 cm to 40.00 cm in animals from 18 to 48 months or more [29]. Usually, taurine (*Bos taurus taurus*) bulls with ages around 12 to 15 months present SC values of 30 cm on average at the beginning of the breeding season. However, Zebu (*Bos taurus indicus*) typically starts puberty around 18 months, with an average SC of 28 cm, indicating slightly lower fertility rates [4]. A study including 609 Nellore bulls reported averages of 12.00 cm for length and 7.00 cm for the width of testicle biometrical measurements [15]. Another study evaluating 15,313 Nellore bulls measured at 22 months, reported averages of 0.36 dm^3^ for TV [27]. These mean values are from young animals and may represent how this is driving selection for testicular biometry in Nellore bulls [30].

Fonseca et al. [22] evaluating 6,162 Nellore bulls for three andrological examination tests, reported averages of 3.01%, 4.63%, and 7.67% for major, minor, and total semen defects, respectively. In the same population, the temporary unfit and culling groups presented semen defects ranging from 8.54% to 39.03%. The identification of suitable bulls for reproduction must consider multiple andrological examinations, combining reproductive efficiency and seminal quality [31].

The genetic material (semen for AI or natural mating bulls) that will be available in the market should contain essential information from semen evaluations, such as viability and sperm morphology, and andrological fitness assessment traits, to ensure high fertilization rates and enable dissemination of genetically-superior bulls [32].

### Female reproductive efficiency and precocity traits

Beef cattle reproduction is more challenging in tropical environments, especially in heifers, as they face adverse environmental conditions and competing energy expenditures for fetal growth, return of a new pregnancy, and lactation [2, 3]. Studies in Nellore cattle reported rebreeding rates ranging from 27.1% to 70.08% [2, 3, 33, 34]. The ability of heifers to conceive a new pregnancy after the first calving is fundamental for the profitability of beef cattle systems. Under this reasoning, ensuring a good energy balance after the first calving, allows the females to raise their calves and be physiologically prepared for the second mating through extended calving rest periods, resulting in higher rates of reproductive success [2].

The ability of females to remain reproductively active in the herd is an important breeding goal for animals to remain in the herd for the long term [35]. STAY can vary across populations depending on the environmental and reproductive management used, as indicated by success rates reported in numerous studies ranging from 25.14% to 41.06% [3, 35]. Regarding female precocity, PP14 is an important breeding goal used by producers aiming to identify females capable of reproducing at a younger age, thus, increasing the profitability of pasture-based herds [36]. A previous study reported a mean success rate of PP14 ranging from 23% to 33.2% between seasons, indicating that in consecutive seasons the daughters of super-early cows, those who had their first calving no later than 27 months, had more than 70% of pregnancy at 14 months compared to non-early progenies [37]. PP14, besides being an easy-to-measure trait, is economically important and should be added to selection indexes aiming to increase sexual precocity in Nellore cattle.

### Heritability estimates for andrological traits

Our results demonstrate that andrological traits have reasonable additive genetic variation and are heritable, indicating that genetic progress for these traits is feasible. The semen quality traits evaluated in this study are lowly to moderately heritable, indicating that in addition to genetic selection, there is a need for improving and better controlling the environmental variability. The main physiological and environmental factors influencing semen quality are puberty, sexual maturation, reproductive hormones, libido, the bull’s health, physical status, and the number of semen collections [4].

Heritability estimates for semen quality traits for ejaculate volume ranged from 0.09 to 0.42, in Hereford herds, multi-breed populations, and Holstein cattle [38–40]. The heritability estimates of sperm vigor and vortex reported in the literature range from 0.05 to 0.07 in Nellore cattle [41–43]. Various studies have been collecting pre/post-thawing MOT data to investigate the mechanisms influencing this trait to improve fertility rates. Heritability estimates ranging from 0.02 to 0.08 and from 0.13 to 0.37 were reported in dairy bulls and multibreed populations, respectively [38, 44]. The differences in breed composition, statistical models, and trait definition might partially explain the difference in the estimates found in this study.

In general, sperm morphological and testicular biometry traits are usually more heritable than semen quality [43]. First, SC is an indirect indicator of precocity in females and, when combined with selection for semen quality traits can improve female reproductive success [28]. The literature emphasizes that SC must be selected in combination with other traits to simultaneously improve precocity in females and males [45, 46]. In addition, SC is essential for sperm thermoregulation, especially for young bulls [47–50]. Several studies have demonstrated high heritability for SC, ranging from 0.43 to 0.63 [27, 43, 51]. SC is one of the most commonly used reproductive traits in beef cattle breeding programs due to its high heritability and genetic relationship with female precocity [52]. It is also important to monitor genetic progress for SC as extreme values might also reduce bull welfare (physical attritions) and fertility rates.

Testicular biometrical traits are related to age at puberty in cattle [53]. Previous Nellore cattle studies have reported high heritability estimates ranging from 0.24 to 0.46 for testicular length and from 0.12 to 0.31 for testicular width [54, 55]. Both traits need to be carefully measured by a well-trained evaluator as they are fundamental for the appropriate calculation of TV and FT [30]. A study of testicular shape showed an average heritability of 0.20 in Nellore bulls [27] and observed changes in testicular shape, from spherical to elongated, which may be related to adaptation in tropical environments, favoring heat exchange as well as TV [30]. A study evaluating TV at three different time points in Nellore cattle reported higher genetic variability and higher heritability estimates as the age advanced from 9 to 12, and to 18 months (0.19, 0.26, and 0.39), respectively [42].

The seminal vesicle produces secretions during ejaculation that provide a healthy environment to maintain sperm quality [56]. Oliveira et al. [54] reported low heritability estimates for seminal vesicle biometry of 0.04 and 0.07 for length and width, corroborating with the results obtained in this study. These results emphasize the high environmental variance related to these traits and indicate that the inclusion of error in the phenotype collection may be affecting the estimates, as they were performed through rectal palpation.

BULL_FIT presented a high heritability (0.69), which is higher compared to literature reports. Previous studies reported heritabilities of 0.10 and 0.12 [27, 51], suggesting that the higher heritability estimates observed in this study might be a result of the BSE classes considered. In contrast, the number of semen straws produced per bull that are suitable for freezing and future distribution for AI had an average heritability of 0.18. This estimate is expected as environmental factors can highly affect the freezing process [44]. The additive genetic variance and heritability estimates observed for all the andrological traits evaluated indicate that they can be genetically improved through direct selection.

### Heritability for female reproductive traits

The rebreeding rate is directly associated with good nutritional status and reproductive management practices, resulting in economic gains from medium to long term through fertility improvement in young females [3]. Thus, the inclusion of a fixed effect in the analysis model for REB, REBB, and REBA, the calving interval rest classes, from the first to the subsequent calving, may result in different heritability estimates in comparison to previous reports. Two previous studies [3, 35] evaluating rebreeding in Nellore cattle based on four classes of rebreeding or based on two classes (failure or success) reported heritability estimates of 0.11 and 0.13, respectively [3, 35].

Low heritability estimates (ranging from 0.05 to 0.08) were also observed for STAY in crossbreed females [20]. In Nellore, heritability estimates of 0.10 for stayability up to 52 months, 0.15 for stayability up to 76 months, [35], and 0.17 for stayability up to 65 months were previously reported [3]. In contrast, a study in Nellore cattle observed a heritability estimate of 0.50 for PP14. In this context, the inclusion of PP14 as a selection criterion in beef cattle breeding programs should increase female precocity over generations [19].

### Genetic correlations among andrological traits

The genetic correlations among the semen quality traits were favorable with moderate to high magnitude, indicating that some genes may simultaneously influence multiple semen quality traits. Butler et al. [57] observed that the attributes of cattle semen are controlled by several QTL associated with fertility in beef cattle. However, we observed high and negative unfavorable correlations between VOL and other semen quality traits, indicating that selection to increase the amount of ejaculate might result in lower sperm quality. This factor may reduce male reproductive performance, as also reported in a study with bulls from five breeds [58]. Furthermore, Gebreyesus et al. [12] reported that the correlated genetic response to VOL would be negative and unfavorable in pre-cryopreservation (-0.53) and post-cryopreservation (-0.54) viability, and another study with pre- and pro-cryopreservation MOT, ranging from -0.11 to -0.17 [44].

Genetic selection for higher SC might not result in higher VOL, which agrees with the literature. On the other hand, favorable genetic correlations were observed between SC and aspects of semen quality, such as SC and VIG (0.56), SC and TURB (0.39), and SC and MOT (0.39). Thus, SC and testicular biometry are considered essential traits due to their easy and inexpensive method of measurement and good correlation with fertility [15, 59].

Regarding the physiological aspects, a viable semen sample should have low to no sperm defects [21]. Semen quality traits showed negative but favorable correlations with sperm defects. As observed in the literature, the genetic correlations between semen quality and sperm defects ranged from -0.34 to -0.75 [40, 60]. MOT is favorably correlated with sperm head and tail defects (-0.71 and -0.78), suggesting that MOT is a good indicator of bull fertility [15, 61].

Studies sought to assess at the end of the BSE, classifications of andrological aspects of the evaluated bulls and considered according to sire aptitude or not, or classified based on breeding type, either natural breeding or the production of viable and non-viable samples and observed high unfavorable correlations between BULL_FIT and SMN_ASPC with VOL. These results indicate that selection for high VOL might generate more inappropriate sires with lower concentration (opalescent) seminal appearance [44, 51]. The results of the genetic correlation between ejaculate volume and concentration (-0.40), VOL with motility after thawing (-0.30), and VOL with rejected semen samples (0.10), indicate that selection for high VOL can reduce seminal viability [38].

Our findings indicate high and favorable genetic correlations between semen quality, sperm concentration, and bull aptitude for reproduction. Bull service rate and the number of straws produced or rejected presented genetic correlations ranging from 0.50 to 0.90, indicating that the selection for one of those traits can also improve the bull's reproductive efficiency [12, 38, 44].

In addition to semen quality, favorable genetic correlations were observed between sperm defects (-0.74) and morphological biometric (0.56) with BULL_FIT, where through BSE results, satisfactory and unsatisfactory bulls were identified [51]. In this sense, the selection for BULL_FIT can indirectly improve the sperm morphological aspects, reducing the percentage of sperm defects and, consequently, increasing the semen quality and fertility of bulls through favorable indirect responses.

### Genetic correlations between female reproductive efficiency and precocity traits

Female fertility traits can be influenced by the same groups of genes modulating the genetic architecture of the Nellore breed. Fertility traits are usually largely influenced by environmental factors and controlled by several genes, which might have pleiotropic effects among traits due to the high and positive genetic correlations observed. Environmental factors highly influence embryonic development and metabolic processes associated with reproductive efficiency and precocity [2, 62, 63]. In this context, high genetic correlations were observed among the female rebreeding classes, indicating that selection for rebreeding could improve REBA and REBB. Females challenged to reproduce at 16 months may have better REB rates with a more extended recovery period between the first and second calving [2]. However, the correlation between PP14 and REBA (0.18) was different compared to the high genetic correlations found between the other breeding categories – PP14 with REB (0.45) and PP14 with REBB (0.47). Selection for heifers challenged at 14 months can generate more productive females in the second calving, despite experiencing a great challenge after the first calving, due to adverse environmental conditions, requiring high nutritional demands, lactation, and development for the next breeding season [3].

One of the main reproduction difficulties in livestock is to challenge females at an early age and remain productive, generating more descendants over generations [64]. In this study we observed high and favorable genetic correlations between rebreeding and STAY. Our results corroborate with previous genetic correlations reported in Nellore cattle for STAY at 76 months (0.98) and STAY at 52 months (0.99) [35]. Selection for female rebreeding tends to result in genetic gains that will impact the success of pregnancy in subsequent calvings. Additionally, the younger a heifer calves, the more efficient she is likely to be during her productive life [20].

### Genetic correlations between female reproductive efficiency and andrological traits

The high favorable correlations observed in this study suggest that the selection of sires with higher genetic merit based on andrological traits could generate precocious females and better annual reproductive performance. In addition, semen quality and testicular biometry can positively affect female fertility and improve the accuracy of genetic assessments [65]. Therefore, the targeted reproduction of genetic materials from bulls with better andrological potential and good semen quality can increase the pregnancy rate and obtain substantial economic gains linked to productive efficiency. Improving the volume and quality of semen from genetically superior bulls can generate a high supply of superior-quality semen, increasing the added value of genetic material available on the market [12, 15]. Thus, selection for bulls with better semen quality based on BSE may significantly impact the reconception of precocious females.

Moderate genetic correlations were observed between morphological traits, such as SC, with early rebreeding. Low and negative genetic correlations were reported for Nellore bulls between female rebreeding and SC measurements at 12 months (-0.13), 15 months (-0.11), and 18 months (0.11) [33]. SC is likely to continue as a key breeding goal to improve female precocity and recording early SC measurements is recommended [66]. No previous reports were found in the literature regarding the moderate genetic correlation between the seminal vesicle and REBA. The seminal vesicles are essential for improving the seminal environment, maintaining motility, and ensuring fertilization [56].

Bulls must have sufficient sperm quality for fertilization. The semen samples need to have low to no defects, mainly with high-quality sperm in the head and tail [61]. Our results showed favorable genetic correlations between rebreeding and STAY traits with sperm defects, suggesting that selection for defect-free semen might improve female reproduction rates. However, to improve the fertility of both bulls and females through correlated responses, there must be a standardization of the phenotypic collection at breeding centers [33].

### Implications and next steps

Our results will inform selection decisions for target traits evaluated in breeding programs, including increasing the quantity and quality of semen produced by high merit bulls. The results presented will also serve as background information for the design of selection indexes, as well as support information for Nellore cattle breeding programs. However, more studies are needed to investigate genes located on autosomal and sex chromosomes that may be associated with these traits. Considering the effects of the sex chromosomes in the field of animal reproduction is important as sex chromosomes contains genes related to development and reproduction.

## Conclusions

Although fertility and reproductive traits have significant environmental influence, the results presented suggest that these traits have genetic variability and when included in selection schemes could contribute to improving genetic gain for both male and female reproductive performance. The selection of bulls for better semen quality, testicular morphology, biometry, and qualification in the andrological examination will enhance the ability of breeding organizations to propagate genetic improvement to the entire population. Selection for female rebreeding will result in more productive females over the years. In addition, improving sperm quality may positively influence genetic progress through correlated responses with fertility and precocity of Nellore females.

## Methods

Animal Care Committee approval was not obtained for this study as all the analyses were performed using pre-existing databases. All the animals included in this study were managed in accordance with the Recommended Code of Practice for the Care and Handling of Farm Animals from the Brazilian Ministry of Agriculture and Livestock (MAPA, Brasilia, DF, Brazil).

### Phenotypic and pedigree datasets

The datasets used were collected in seedstock Nellore cattle farms (Agro-Pecuária CFM, São José do Rio Preto, SP, Brazil) located in the Brazilian states of São Paulo and Mato Grosso do Sul. These datasets are managed by the Center for Research in Animal Improvement, Biotechnology, and Transgenics (GMABT) at the University of Sao Paulo (USP, Pirassununga, SP, Brazil). The pedigree dataset contained 660,608 animals spanning up to eight equivalent generations. The phenotypic datasets included records from heifers, cows, and bulls collected from 14 to 48 months of age. These animals were born between 1999 and 2020.

The animals were raised in management groups under grazing systems in cultivated tropical pastures composed of approximately 40% *Brachiaria brizantha*, 50% *Panicum maximum*, and 10% of other grasses, and supplemented with a mineral mixture. All the animals were vaccinated and treated for diseases as needed based on recommendations of the veterinarian and sanitary defense agencies of their respective regions.

The female traits evaluated in this study were: probability of pregnancy at 14 months (PP14), ability to remain productive in the herd at least until four years, producing one calf per year (STAY), and female re-breeding, including general rebreeding of females throughout their lives (REB), rebreeding of females that start reproduction at two years old (REBB), and re-breeding of heifers, up to 14 months (REBA).

The male traits evaluated can be defined based on the following groups: 1) semen quality traits: ejaculate volume (VOL, in mL), vortex (TURB, scale from 0 to 5), rectilinear progressive sperm motility (MOT, in %), and spermatic vigor (VIG, scale from 0 to 5); 2) morphological traits: scrotal circumference (SC, in cm), testicular format (TF, in cm), left and right testicular length (LTL and RTL, in cm), left and right testicular width (LTW and RTW, in cm), testicular volume (TV, in dm^3^), and seminal vesicle width (VESICW, in cm) and length (VESICL, in cm); 3) sperm defects: total sperm defects (TD, in %), total minor defects (MID, in %), and major defects (MAD, in %); and 4) overall semen evaluation: andrological fitness (BULL_FIT, scale from 1 to 4) and seminal aspects (SMN_ASPC, scale from 1 to 4). A detailed description of the phenotypic measurements and trait definition are provided below.

The PP14 trait was measured about 60 days after the end of the breeding season. Heifers (exposed to a bull at about 14 months of age, range 12–16 months) from three farms were submitted to rectal palpation or ultrasound for the diagnosis of pregnancy. Heifer pregnancy (HP) was analyzed as a categorical trait, with a value of “2” (success) being assigned to heifers that were diagnosed pregnant and a value of “1” (failure) being assigned to those that were not pregnant at that time [36]. STAY was defined as the ability of the cow to remain productive in the herd for four years or more, producing one calf per year, in which “1” indicates failure and “2” indicates success [67]. The score “2” was assigned to cows that were at least four years old and had one calf per year. The rebreeding traits indicate whether the female became pregnant in the previous breeding [68]. All three traits are binary, in which a success (2) or failure (1) record was attributed to the females at the end of the breeding season.

The breeding soundness evaluation (BSE) was carried out by a single evaluator (Prof. Dr. José Domingos Guimarães) in June and July of each year from 1998 to 2020. Semen samples were collected using the electro-ejaculation method [69], and the physical and morphological characteristics of the sperm were evaluated according to the methodology suggested by Henry and Neves [70]. SC was measured using a tape in the wider region of the scrotum after gonads ventrocaudal traction. SC was measured in animals at approximately 18 months of age. The physical and morphological examinations were part of the andrological assessment. The biometric testicular measurements (LTL, RTL, LTW, RTW) were done using a caliper during the andrological examination at approximately 18 months of age together with the SC evaluation. VESICL and VESICW were also measured at the same time through transrectal palpation and quantification of the width and length of the seminal vesicles.

The testicular format (TF) was defined as long-moderate, long-ovoid, ovoid-spherical, and spherical [47, 71] The classes were defined through the ratio between the mean LTW and RTW and the mean LTL and RTL on a scale of 0.5 to 1.0, where 0.5 mean a width equal to half the length and 1.0 a width equal to the length. For each TF class, threshold values were established as follows: 1 = long – ratio ≤ 0.5, 2 = long-moderate – ratio from 0.51 to 0.625; 3 = long-oval – ratio from 0.626 to 0.750; 4 = oval-spherical – ratio from 0.751 to 0.875; and, 5 = spherical – ratio > 0.875. TV was calculated as: 2[(r^2^) x π x L], where: r = radius (testicular width); π = correction factor (3.14); and L = testicular length [72].

A drop of semen from each ejaculate was placed on a slide, previously heated to 37 °C, for observation of TURB (scale from zero to five) under an optical microscope at 100 × magnification. An additional drop of semen was placed between the slide and coverslip, previously heated to 37ºC, to evaluate MOT (expressed in percentage) and VIG (scale from zero to five), with an increase of 100 to 400 times. Part of the ejaculate aliquot was utilized to evaluate SMN_ASPC with four classes: creamy (1), milky (2), watery (3), and opalescent (4). An aliquot from the ejaculate was diluted in buffered saline formaldehyde solution and utilized in the sperm morphology analyses [73].

In phase-contrast microscopy, the sperm pathology was evaluated in moist preparations, between a slide and coverslip, at a magnification of 1,250 times under an immersion objective lens. In each sample, 400 cells were evaluated, and the percentage of normal sperm and anomalies of the acrosome, head, intermediate piece, and tail were determined [74]. Later, the semen defects were classified according to Henry and Neves [70]. TD, MID, and MAD were considered indicators of sperm morphology. At the end of the andrological evaluation, an andrological fitness test was performed where the bull received (BULL_FIT) a value of 1: able to become semen donor or natural mating bull; 2: exclusively suitable for natural matings; 3: temporarily unfit bull with restriction of breeding (recommendation of future re-evaluation); and, 4: unfit, andrological pattern of sire's fitness for reproduction.

Contemporary groups (CGs) were created considering animals born on the same farm, year and season of birth, of the same sex, and belonging to the same management group, for both, male and female traits, except for SC and PP14 in which management group was an uncorrelated random effect. Additionally, only records within ± 3.5 standard deviations from the CG mean were maintained for further analyses. CG with less than five animals and without phenotypic variability were excluded. Any CG with progeny from less than two bulls or formed by animals with unknown pedigree was also removed.

### Genomic datasets

A total of 7,975 animals were genotyped using the GeneSeek SNP Beadchip Bovine GGP-HDi 50 K (54,701 SNPs). The quality control (QC) was performed using the PREGSf90 package [75]. Animals and markers with a call rate lower than 90% were removed from further analyses. Genotyped animals with more than 1% parent-progeny Mendelian conflicts were removed. Furthermore, SNPs with minor allelic frequency (MAF) lower than 0.05, extreme deviations from Hardy–Weinberg equilibrium defined by the maximum difference between the observed and expected frequency of heterozygosity higher than 0.15 [76], duplicated or unknown position, and those located in non-autosome chromosomes were also removed. After QC, 7,303 genotyped animals and 44,135 SNPs remained for further analyses.

### Statistical analyses

#### Estimation of genetic parameters

The single-step genomic BLUP (ssGBLUP) procedure based on Bayesian inference was used for all traits. The ssGBLUP is a modification of the BLUP model, where the inverse of the pedigree relationship matrix (**A**^−1^) is replaced by the **H**^−1^ matrix [77], as follows:

$${{\varvec{H}}}^{-1}={{\varvec{A}}}^{-1}+\left[\begin{array}{cc}0& 0\\ 0& {{\varvec{G}}}^{-1}-{{\varvec{A}}}_{22}^{-1}\end{array}\right]$$

where **H** is a combined relationship matrix of genotyped and non-genotyped individuals; **A** is the pedigree-based relationship matrix; **A**_22_ is a numerator relationship matrix for the genotyped animals, and **G** is the genomic relationship matrix calculated as [78]:

$${\varvec{G}}={\varvec{Z}}{{\varvec{Z}}}^{\boldsymbol{^{\prime}}}$$

where, **Z** is the matrix containing adjustments for allelic frequencies. These factors are fitted to ensure that the mean diagonal of **G** is close to **A**_22_ [79].

The models used to estimate the variance components and genetic parameters are shown in Table 4. For REB, REBB, and REBA, the model included the calving rest interval (CRI—in days) of the females, that is, the days postpartum until the beginning of the second mating: < 60 days, > 61 to < 90 days, > 91 to < 120 days, > 121 to < 150 days, and > 151 days. For all models **a** was considered the random animal effect; and **e** represents the residual random terms. For the estimates of genetic correlations, a bi-trait model was used:

$$\left[\begin{array}{c}{{\varvec{y}}}_{1}\\ {{\varvec{y}}}_{2}\end{array}\right]= \left[\begin{array}{c}{{\varvec{X}}}_{1}\\ 0\end{array} \begin{array}{c}0\\ {{\varvec{X}}}_{2}\end{array}\right] . \left[\begin{array}{c}{{\varvec{\upbeta}}}_{1}\\ {{\varvec{\upbeta}}}_{2}\end{array}\right]+\left[\begin{array}{c}{{\varvec{Z}}}_{1}\\ 0\end{array}\begin{array}{c}0\\ {{\varvec{Z}}}_{2}\end{array}\right] . \left[\begin{array}{c}{{\varvec{a}}}_{1}\\ {{\varvec{a}}}_{2}\end{array}\right]+ \left[\begin{array}{c}{{\varvec{e}}}_{1}\\ {{\varvec{e}}}_{2}\end{array}\right]$$

For the categorical traits, a priori, the distributions of vectors **y**, **a**, and **e**: $\mathbf{y}\sim \mathrm{MVN}\left(\mathbf{X}{\varvec{\upbeta}}+\mathbf{Z}\mathbf{a},\mathbf{R}\right)$, $\mathbf{a}|\mathbf{H},{\mathbf{G}}_{\mathrm{a}}\sim \mathrm{MVN}\left(0,\mathbf{H}\otimes {\mathbf{G}}_{\mathrm{a}}\right)$, and $\mathbf{e}|\mathbf{I},\mathbf{R}\sim \mathrm{MVN}\left(0,\mathbf{I}\otimes \mathbf{R}\right)$; where: **H** is the relationship matrix considering all animals included in the analyses (genotyped and non-genotyped); **R** is a residual (co)variance matrix; **I** is an identity matrix of proper order; **G**_a_ is the additive genetic (co)variance matrix, and ⨂ is the Kronecker product. For the systematic effects, a uniform a priori distribution was defined. A single Markov Chain Monte Carlo (MCMC) was generated with 800,000 samples, and the first 200,000 samples were discarded as burn-in. The remaining samples were saved in a range of 100 samples. Consequently, the inferences were made based on 6,000 samples from the posterior distribution of the parameters. The convergence was evaluated using the Geweke test [80], and boxplot and heatmaps to illustrate genetic correlations obtained through the R software.

A threshold animal model considering the Probit link function was used to estimate the variance components and genetic parameters for the categorical traits using the BLUPf90 + family programs [81]. Alpha (0.90) and beta (0.10) parameters were used to construct the **H** matrix. For the genetic correlations, the Gibbs sampling method was applied using the software GIBBS1F90 or THRGIBBS1F90 [82] for linear or categorical traits, respectively. For all the heritability and genetic correlation estimates, their respective standard errors (SE) were calculated using the standard deviation ($\sigma$) of their sample distribution divided by the square root of the number of records (N) present in a given dataset: SE = $\frac{\sigma }{\sqrt{N}}$. 

## Supplementary Information

**Additional file 1: Figure S1.** Boxplot of calving rest interval (CRI) of REB: All records of rebreeding of females; REBB: Rebreeding of females that entered reproduction at two years old; REBA: Rebreeding of precocity heifers; IR: Interval of rest (in days); N: Number of animals in each class.

## Data Availability

All the data supporting the results of this article are included within the article and in its supplementary files. The raw data cannot be made publicly available, as it is the property of some Brazilian Nellore breeding companies and this information is commercially sensitive. Access to the raw datasets for research purposes can be made to Dr. Jose B. S. Ferraz (jbferraz@usp.br).

## Abbreviations

AI: Artificial insemination
BSE: Breeding soundness exam
BULL_FIT: Andrological fitness
CG: Contemporary group
EBV: Estimated breeding value
GMAND: Management group
HP: Heifer pregnancy
LTL and RTL: Left and right testicular length
LTW and RTW: Left and right testicular width
MAD: Major defects
MID: Total minor defects
MOT: Rectilinear progressive sperm motility
PP14: Probability of pregnancy at 14 months
QC: Quality control
REB: Female rebreeding
REBA: Rebreeding of heifers, up to 14 months
REBB: Rebreeding of females that start reproduction at two years old
STAY: Stayability
TD: Total sperm defects
TF: Testicular format
TV: Testicular volume
TURB: Vortex
SC: Scrotal circumference
SMN_ASPC: Seminal aspects
SNP: Single nucleotide polymorphism
VESICW: Seminal vesicle width
VESICL: Seminal vesicle length
VIG: Spermatic vigor
VOL: Ejaculate volume
