# Supplementary material for: Genetic parameters for various semen production and quality traits and indicators of male and female reproductive performance in Nellore cattle
Source: BMC Genomics. 2023 Mar 27;24:150. doi: 10.1186/s12864-023-09216-5 (PMC10044441; doi:10.1186/s12864-023-09216-5)
Supplement: Supplementary file 1 — Additional file 1: Figure S1. Boxplot of calving rest interval (CRI) of REB: All records of rebreeding of females; REBB: Rebreeding of females that entered reproduction at two years old; REBA: Rebreeding of precocity heifers; IR: Interval of rest (in days); N: Number of animals in each class. [file 12864_2023_9216_MOESM1_ESM.docx]

**SUPPLEMENTARY MATERIAL**


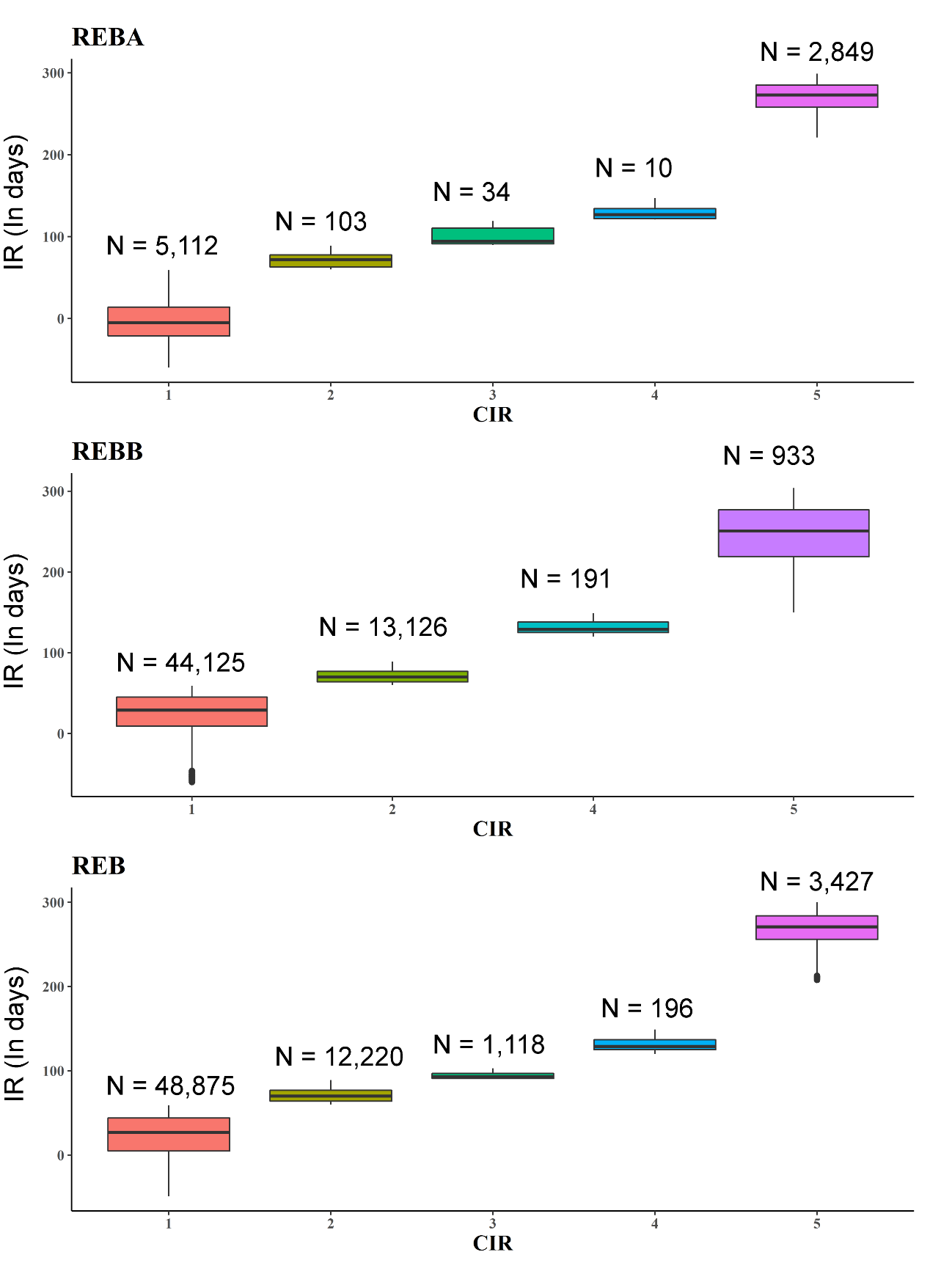
 **Figure S1.** Boxplot of calving rest interval (CRI) of REB: All records of rebreeding of females; REBB: Rebreeding of females that entered reproduction at two years old; REBA: Rebreeding of precocity heifers; IR: Interval of rest (in days); N: Number of animals in each class.
